# A Novel Tool to Predict the Overall Survival of High-Grade Osteosarcoma Patients after Neoadjuvant Chemotherapy: A Large Population-Based Cohort Study

**DOI:** 10.1155/2022/8189610

**Published:** 2022-07-23

**Authors:** Zhangheng Huang, Yu Wang, Ye Wu, Chuan Guo, Weilong Li, Qingquan Kong

**Affiliations:** Department of Orthopedics, Orthopedic Research Institute, West China Hospital, Sichuan University, Chengdu, Sichuan, China

## Abstract

**Background:**

The goal of this study was to discover clinical factors linked to overall survival in patients with high-grade osteosarcoma who had received neoadjuvant therapy and to develop a prognostic nomogram and risk classification system.

**Methods:**

A total of 762 patients with high-grade osteosarcoma were included in this study. In the training cohort, Cox regression analysis models were used to find prognostic variables that were independently linked with overall survival. To predict overall survival at 3, 5, and 8 years, a nomogram is created. In addition, in both the internal and external validation cohorts, receiver operating characteristic curves, calibration curves, and decision curve analysis (DCA) were utilized to assess the prediction model's performance.

**Results:**

The age, size of the tumor, and the stage of the disease are all important predictive variables for overall survival. The training and validation cohorts have C-indexes of 0.699 and 0.669, respectively. At the same time, the area under the curve values for both cohorts also showed that the nomogram had good discriminatory power. The calibration curve demonstrated the good performance and predictive accuracy of the model. The DCA results suggest that the nomogram has a wide range of therapeutic applications. Furthermore, a new risk classification system based on the nomogram was established, which allows all patients to be classified into three subgroups as high, middle, and low risk of death.

**Conclusion:**

The prognostic nomogram constructed in this study may provide a better precise prognostic prediction for patients with high-grade osteosarcoma after neoadjuvant chemotherapy.

## 1. Introduction

Osteosarcoma is a common threatening bone cancer that happens predominantly in children and adolescents [1]. At the time of diagnosis, about 90% of these individuals are diagnosed with high-grade osteosarcoma [2]. Osteosarcoma is highly aggressive, which makes the clinical prognosis for patients often poor [3,4]. In recent years, there have been advances in the treatment of osteosarcoma, which have had a positive impact on the prognosis of patients. For high-grade osteosarcoma, neoadjuvant chemotherapy combined with excision of the primary tumor is currently the standard treatment [5]. Although the implementation of neoadjuvant chemotherapy has gradually improved the overall survival (OS) rate of patients, there are still 20–30% of patients whose survival rate has not improved significantly due to the presence of drug-resistant tumor cells [5,6]. Despite the great success of osteosarcoma treatment, there has been a plateau in patient prognosis, i.e., there has been no sustained improvement in osteosarcoma survival rates. Therefore, there is a pressing have to explore and discover prognostic factors associated with patients with high-grade osteosarcoma following neoadjuvant chemotherapy and surgery. Subsequently, this can inform clinical decision-making to improve the treatment outcomes and survival outcomes of high-grade osteosarcoma patients.

Traditionally, clinicians have used TNM staging or Enneking staging to assess the prognosis of patients with osteosarcoma [7]. Unfortunately, even when patients are in the same TNM stage or Enneking stage, their prognosis often varies, suggesting that existing staging systems may be inadequate for making treatment decisions and assessing prognosis. The nomogram is a reliable predictive tool that incorporates various risk factors to evaluate an individual's survival outcome and is represented visually in a graphical format [8–11]. The advent of nomograms has fulfilled the requirement for an integrated model and has played a significant role in driving personalized medicine [10]. Given the heterogeneity of high-grade osteosarcomas, there is an urgent need for a validated predictive model as a tool to personalize and predict the prognosis in patients with high-grade osteosarcoma following neoadjuvant chemotherapy. Therefore, this study aimed to retrospectively analyze data from the Surveillance, Epidemiology, and End Results (SEER) database on high-grade osteosarcoma patients who received neoadjuvant chemotherapy and surgery to explore their clinical characteristics and determine their prognostic factors. The findings of the research will be given access to physicians in the form of a clinically useable nomogram and risk classification system to avoid over or under-treatment and inform clinicians in the development of treatment plans.

## 2. Materials and Methods

### 2.1. Patients Population

The SEER database is the biggest publicly available database of cases from 18 cancer registries in the US [12,13]. The SEER database was accessed via the SEER*∗*Stat software to acquire data on osteosarcoma patients between 2004 and 2015. The inclusion criteria for the data were as follows: pathologically confirmed osteosarcoma with the only primary tumor, high-grade tumor grading (grade III or IV), and patients received neoadjuvant chemotherapy and resection of the primary tumor. Exclusion criteria are as follows: OS < 1 month, patients with missing clinicopathological features, and patients in whom osteosarcoma is not the only primary tumor. Because of the retrospective nature of this study and the anonymity of the patient's data, informed permission was not necessary. Ultimately, we selected 762 patients with high-grade osteosarcoma who had received neoadjuvant chemotherapy and surgery.

### 2.2. Clinicopathological Data

Relevant information included in this study had demographic information, tumor characteristics (disease stage, laterality, tumor size, primary site, tumor grade), and treatment information. X-tile software was used to estimate the appropriate cutoff value for age and tumor size in terms of OS [14]. Subsequently, age was divided into <16 years, 16–21 years, and >21 years. The tumor size was divided into <64 mm, 64–139 mm, and >139 mm. Localized (defined as tumor limited to the periosteum), regional (defined as tumor beyond the periosteum without distant metastases), and distant (defined as tumor beyond the periosteum with distant metastases) are the three types of disease stages [15,16]. In the current study, we defined preoperatively received chemotherapy as neoadjuvant chemotherapy. The selection and definition of survival endpoints play a critical role in cancer-related research. The primary outcome in this research was OS, which was defined as the time from diagnosis to death (from any cause).

### 2.3. Statistical Analysis

In a 7 : 3 ratio, all patients in the study were randomly assigned to the training and validation cohorts. All variables were analyzed independently using univariate Cox regression models. Those variables that proved to have a significant effect on survival were included in the multivariate Cox regression analysis. The Cox proportional risk hypothesis was tested using Kaplan–Meier survival curve analysis. The RMS package in R software was used to create a nomogram. The discriminatory ability of the nomogram was evaluated by the area under the receiver operating characteristic curves and the C-index, and the goodness of fit between the predicted and observed values was assessed by the calibration curve. C-index and area under the curve (AUC) values range between 0.5 and 1.0, with a C-index and AUC value greater than 0.7 usually indicating a reasonable estimate. The nomogram's clinical benefit and utility were assessed using decision curve analysis (DCA) [17]. The curves for the treat-all patient's scenario (representing the highest clinical benefit) and the no treatment scenario (representing no clinical benefit) were also plotted as two references. The nomogram calculates the patient's total score and uses the X-tile software to select the cutoff point for the risk of death stratification. Based on this, a mortality risk classification system was constructed to classify the mortality risk of high-grade osteosarcoma patients who had received neoadjuvant chemotherapy and surgery into three subgroups. Meanwhile, the log-rank test and Kaplan–Meier survival curve analysis were performed to analyze the differences in overall survival between the three subgroups to confirm the nomogram's predictive usefulness. All statistical analyses were carried out using SPSS 25.0 and R (https://www.r-project.org), with a *p* value of less than 0.05 being considered statistically significant.

## 3. Results

### 3.1. Patients Characteristics

762 eligible patients in total were identified from the SEER database and categorized into the SEER training cohort (*n* = 534) and the SEER internal validation cohort (*n* = 228).  Table 1 provides the demographic and clinical characteristics of the patients in the two groups. In the training cohort, there were 289 cases (54.1%) aged <16 years, 136 cases (25.5%) aged 16–21 years, and 109 cases (20.4%) aged >21 years. There were 303 (56.7%) males and 231 (43.3%) females. Of these, 401 (75.1%) were white, 89 (16.7%) were black, and 44 (8.2%) were others. Regional tumors (276 cases, 51.7%) and localized tumors (147 cases, 27.5%) were more common in these high-grade osteosarcomas, and tumor metastases occurred in 111 (20.8%) patients.

### 3.2. Nomogram Variable Screening

**Figure 1:**
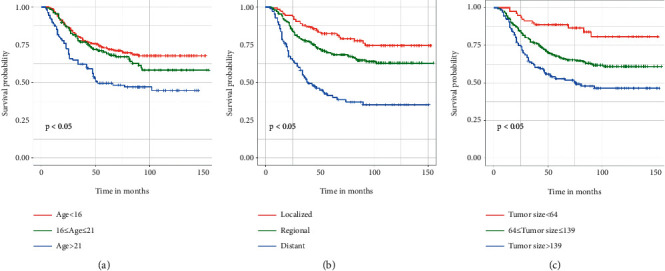
Kaplan–Meier survival curves of variables were performed for high-grade osteosarcoma patients after neoadjuvant chemotherapy and primary tumor resection in the training cohort. (a) Age. (b) Disease stage. (c) Tumor size.

A total of 11 variables were analyzed, and six factors (disease stage, postoperative chemotherapy, tumor size, age, primary tumor site, and marital status) were found to be related to OS (Table 2). To control confounding variables, the risk factors identified by univariate Cox analysis were further explored in a multivariate Cox analysis. The results of multivariate Cox regression analysis showed that disease stage (distant, HR = 4.145, 95% CI = 2.670–6.436, *P* < 0.001), tumor size (>139 mm, HR = 3.134, 95% CI = 1.680–5.881, *P* < 0.001), and age (>21 years, HR = 2.597, 95% CI = 1.838–3.670, *P* < 0.001) were independent prognostic factors affecting OS. In contrast, postoperative chemotherapy, tumor size, and marital status were not statistically significant (Table 2). The Kaplan–Meier survival analysis revealed that clinical factors (age, disease stage, and tumor size) were found to be strongly linked with OS, further validating the results of multivariate Cox regression analysis (Figure 1).

### 3.3. Construction and Validation of a Nomogram

**Figure 2:**
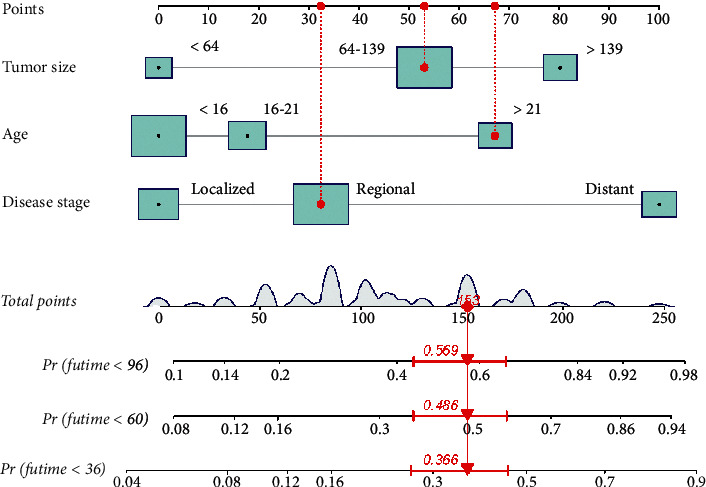
A prognostic nomogram for high-grade osteosarcoma patients after neoadjuvant chemotherapy.

**Figure 3:**
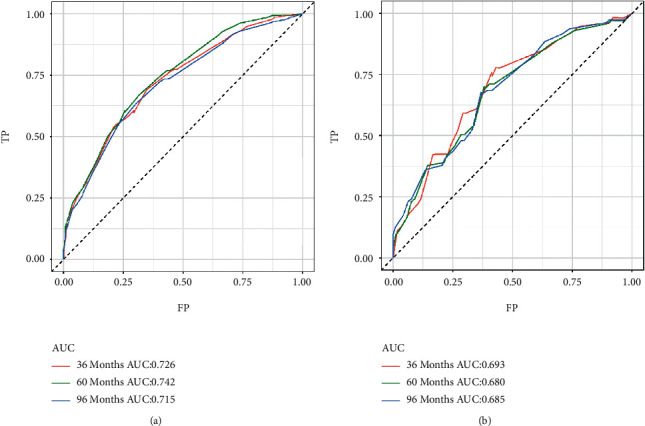
ROC curves and AUC of nomograms for predicting OS at 3, 5, and 8 years in the training cohort (a). ROC curves and AUC of nomograms for predicting OS at 3, 5, and 8 years in the validation cohort (b). ROC, receiver operating characteristic; AUC, area under the curve; OS, overall survival.

**Figure 4:**
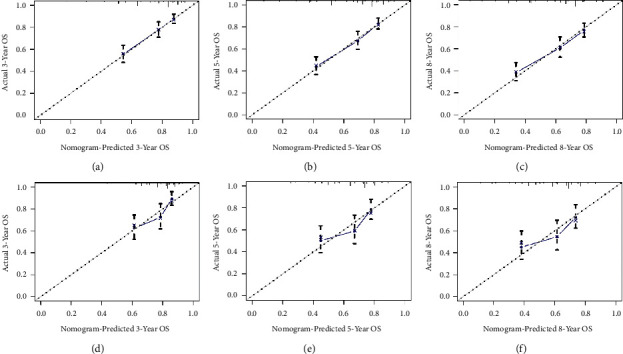
Calibration curves for OS at 3 years (a), 5 years (b), and 8 years (c) for high-grade osteosarcoma patients after neoadjuvant chemotherapy and primary tumor resection in the training cohort. Calibration curves for OS at 3 years (d), 5 years (e), and 8 years (f) in the validation cohort of patients with high-grade osteosarcoma after neoadjuvant chemotherapy and primary tumor resection in the validation cohort. OS, overall survival.

**Figure 5:**
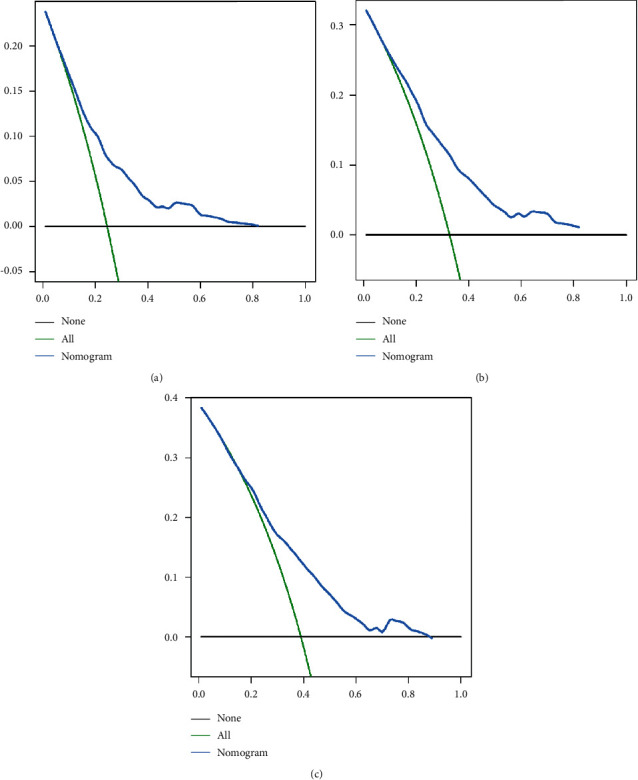
Decision curve analysis depicting the net clinical benefit of OS at 3 (a), 5 (b), and 8 (c) years for the training cohort. OS, overall survival.

**Figure 6:**
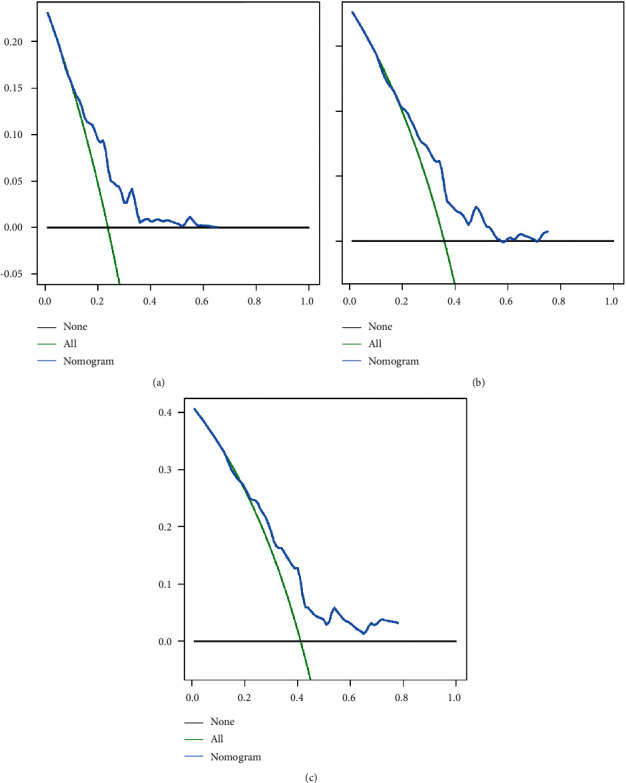
Decision curve analysis depicting the net clinical benefit of OS at 3 (a), 5 (b), and 8 (c) years for the validation cohort. OS, overall survival.

To explore a quantitative method of predicting OS at 3, 5, and 8 years, we developed a prognostic nomogram based on three predictive variables (Figure 2). Figure 2 shows an example of using a nomogram to predict a given patient's overall probability of survival. The probability of patient survival is determined by adding the corresponding scores for each selected variable to obtain a total score and drawing a vertical line from the total score to the time axis. In this study, the majority of patients had a total score between 0 and 250. To facilitate the use of the nomogram model in the clinical work of clinicians who are not adept at nomograms, we also created a web-based nomogram to assess the overall survival of patients with osteosarcoma who received neoadjuvant chemotherapy (https://hzhorthopaedics.shinyapps.io/Web-based nomogram/). To verify the model's predictive accuracy, we evaluated it using the C-index and AUC. The C-index values were 0.699 and 0.669 for the training and validation cohorts, respectively. The AUC values predicted in the training cohort were 0.726, 0.742, and 0.715 at 3, 5, and 8 years, respectively. The AUC values in the validation cohort were 0.693, 0.680, and 0.685, respectively (Figure 3). Bootstrap resampling was used 1000 times to depict the calibration curves for both cohorts. As shown in Figure 4, there was a high agreement between the observed OS probabilities and the OS probabilities predicted using the model. DCA for both cohorts revealed that the model provided a more significant net benefit than the “all treatment” or “no treatment” strategy across a wide range of mortality risks, indicating the potential clinical efficacy of this nomogram (Figures 5 and 6).

### 3.4. Risk Stratification Based on the Nomogram

**Figure 7:**
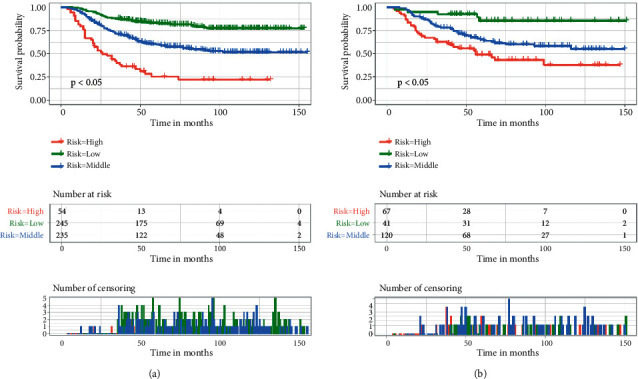
Kaplan–Meier survival analysis and log-rank tests used to compare OS in the low-risk, middle-risk, and high-risk groups of all patients in the training cohort (a) and validation cohort (b). OS, overall survival.

The X-tile software revealed that the best cutoff values for the total mortality risk score were 100 and 179 and were used for risk stratification. Patients were divided into three mortality risk subgroups: low-risk (total score <100), middle-risk (100 ≤ total scores ≤179), and high-risk (total score >179). Kaplan–Meier survival curves and log-rank tests were used to validate the mortality risk stratification. The results showed significant differences in the probability of survival between risk subgroups (Figure 7, *p* < 0.05). The above results suggest that the risk classification system constructed based on nomogram can effectively differentiate patients at high risk of death among patients.

## 4. Discussion

High-grade osteosarcoma is an extremely rare malignant bone tumor compared to some common cancers such as breast cancer and lung cancer. High-grade osteosarcoma has a high propensity for pulmonary metastases and poor prognosis when treated with surgery alone without chemotherapy (2-year survival rate <20%) [18]. The 5-year survival rate for individuals with high-grade osteosarcoma has increased to 60%, thanks to developments in multimodal therapy, notably neoadjuvant chemotherapy [4,19]. Neoadjuvant chemotherapy eliminates early micrometastases and eliminates the primary lesion, thereby reducing adhesions between the tumor and surrounding tissue [20]. Neoadjuvant chemotherapy combined with surgery has been adopted as the main treatment strategy for high-grade osteosarcoma [5,21]. In clinical practice, it is difficult to predict the OS of a particular patient accurately, yet personalized medicine is playing an increasingly important role in cancer treatment. Given the heterogeneity and rarity of high-grade osteosarcoma, this study developed and validated a nomogram and risk stratification system for predicting OS by using the SEER database, which includes 28% of the US population. The development of predictive models can help avoid over or under-treatment and help clinicians develop treatment strategies earlier, thus benefiting both clinicians and patients.

This study showed that as age increased, the survival outcome of patients became worse. Patients aged >21 years (hazard ratio = 2.597, 95% confidence interval = 1.838–3.670) had a higher risk of death and a worse prognosis than the <16 years and 16–21 years age groups. We believe this may be because older patients are more likely to develop metastatic disease and receive chemotherapy less frequently or at lower doses, resulting in a poorer prognosis [22–24]. In addition, the physical developmental status may also contribute to this phenomenon, as human aging is accompanied by cellular senescence, including nuclear genomic instability, protein, and metabolic changes [25,26]. There is no consensus in previous studies on whether tumor size affects the OS of osteosarcoma patients. It has been suggested that the size of the tumor does not matter as small tumors can be aggressive, while other studies have shown that the size of the tumor affects the prognosis of patients with osteosarcoma [27,28]. In our analysis, large tumors consistently predicted a poor prognosis, whereas axial tumors did not affect the prognosis of patients. Larger tumors predispose patients to metastases during and after treatment and pose a significant challenge to clinicians in achieving complete tumor removal [16]. With the advent of neoadjuvant chemotherapy, there has been a marked improvement in survival rates for high-grade osteosarcoma patients. However, Kansara et al. reported that many patients develop metastases at initial diagnosis, with less than 20% of them surviving for more than five years [29]. Once a patient has metastasized, the prognosis is poor, probably because patients who have metastasized tend to be resistant to intensive treatment. In addition to this, patients with regional osteosarcoma showed a worse prognosis than those with localized osteosarcoma after controlling for confounding factors by multivariate Cox regression analysis.

The predictive model constructed allows surgeons to efficiently and accurately predict the overall mortality of individual patients and stratify the risk of death for patients. Overall, the nomogram and risk stratification system we have constructed so far offers the possibility to break the treatment plateau and further improve the prognosis of high-grade osteosarcoma after neoadjuvant chemotherapy and surgery. Ultimately, it is undeniable that there are still some limitations to this study. First, this study is limited by the fact that because our study is retrospective, some data on patients will inevitably be lost, and this may be subject to selection bias. Second, large randomized controlled trials and multicenter clinical samples need to be applied to validate the performance and reliability of the nomogram model. Although, the prediction model we constructed did not achieve very precise prediction accuracy. However, for clinicians and patients, the model we constructed can be used as a reference and provide the possibility to improve the prognosis of patients. Meanwhile, we hope that soon, we can add more variables and patient information (e.g., tumor markers and gene expression variables) to this foundation based on the current study to develop a more comprehensive and superior prediction model.

## 5. Conclusions

Based on three independent prognostic factors screened, we developed a nomogram and risk stratification system for high-grade osteosarcoma patients who received neoadjuvant chemotherapy. In both internal and external cohorts, the model has performed well, allowing clinicians to utilize it as a reference tool for making clinical choices and stratifying patient care. It also gives clinicians a starting point for determining suitable stratification parameters in future clinical trials.

## Figures and Tables

**Table 1:** Clinical and pathological characteristics of high-grade osteosarcoma patients after neoadjuvant chemotherapy.

Variables	Total cohort		Training cohort		Validation cohort	
	*N* = 762		*N* = 534		*N* = 228	
	*n*	%	*n*	%	*n*	%
Age						
<16	409	53.7	289	54.1	120	52.6
16–21	194	25.5	136	25.5	58	25.4
>21	159	20.9	109	20.4	50	21.9
Race						
Black	124	16.3	89	16.7	35	15.4
White	568	74.4	401	75.1	167	73.2
Others	79	10.4	44	8.2	26	11.4
Sex						
Male	431	56.6	303	56.7	128	56.1
Female	331	43.3	231	43.3	100	43.9
Primary site						
Upper limb	114	15.0	77	14.4	37	16.2
Lower limb	613	80.4	436	81.6	177	77.6
Others	35	4.6	21	3.9	14	6.1
Grade						
III	266	34.9	191	35.8	75	32.9
IV	496	65.1	343	64.2	153	67.1
Laterality						
Left—origin of primary	389	51.0	263	49.3	126	55.3
Right—origin of primary	373	49.0	271	50.7	102	44.7
Radiotherapy						
No	742	97.4	519	97.2	223	97.8
Yes	20	2.6	15	2.8	5	2.2
Postoperative chemotherapy						
No	279	36.6	192	36.0	87	38.2
Yes	483	63.4	342	64.0	141	61.8
Disease stage						
Localized	226	29.7	147	27.5	79	34.6
Regional	378	49.6	276	51.7	102	44.7
Distant	158	20.7	111	20.8	47	20.6
Tumor size						
<64	110	14.4	79	14.8	31	13.6
64–139	465	61.0	327	61.2	138	60.5
>139	187	24.5	128	24.0	59	25.9
Marital status						
Unmarried	689	90.4	481	90.0	208	91.2
Married	73	9.6	53	10.0	20	8.8

**Table 2:** Analysis of univariate and multivariate Cox regression in high-grade osteosarcoma patients after neoadjuvant chemotherapy.

Characteristics	Univariate analysis		Multivariate analysis	
	HR (95% CI), *P* value		HR (95% CI), *P* value	
Age				
<16	Reference		Reference	
16–21	1.238 (0.869–1.766)	0.237	1.287 (0.899–1.841)	0.168
>21	2.140 (1.526–3.002)	<0.001	2.597 (1.838–3.670)	<0.001
Race				
Black	Reference			
White	0.880 (0.604–1.281)	0.504		
Others	1.120 (0.632–1.983)	0.698		
Sex				
Male	Reference			
Female	0.808 (0.603–1.082)	0.152		
Primary site				
Upper limb	Reference			
Lower limb	0.655 (0.452–0.948)	0.025		
Others	1.401 (0.711–2.759)	0.330		
Grade				
III	Reference			
IV	0.867 (0.646–1.164)	0.342		
Laterality				
Left—origin of primary	Reference			
Right—origin of primary	1.080 (0.811–1.438)	0.600		
Radiotherapy				
No	Reference			
Yes	1.594 (0.749–3.394)	0.226		
Postoperative chemotherapy				
No	Reference			
Yes	0.730 (0.546–0.975)	0.033		
Tumor size				
<64	Reference		Reference	
64–139	2.596 (1.432–4.708)	0.002	2.135 (1.168–3.905)	0.014
>139	4.208 (2.269–7.804)	<0.001	3.134 (1.680–5.881)	<0.001
Disease stage				
Localized	Reference		Reference	
Regional	1.687 (1.122–2.536)	0.012	1.587 (1.051–2.396)	0.028
Distant	4.141 (2.699–6.353)	<0.001	4.145 (2.670–6.436)	<0.001
Marital status				
Unmarried	Reference			
Married	1.620 (1.063–2.469)	0.025		

## Data Availability

The data used to support this study were analyzed in this study and are available at SEER dataset repository (https://seer.cancer.gov/).

## Abbreviations

SEER: Surveillance, Epidemiology, and End Results
OS: Overall survival
AUC: Area under the curve
DCA: Decision curve analysis.
